# Differentially expressed genes in the caecal and colonic mucosa of Landrace finishing pigs with high and low food conversion ratios

**DOI:** 10.1038/s41598-017-14568-6

**Published:** 2017-11-02

**Authors:** Zhen Tan, Yuan Wang, Ting Yang, Kai Xing, Hong Ao, Shaokang Chen, Fengxia Zhang, Xitong Zhao, Jianfeng Liu, Chuduan Wang

**Affiliations:** 1 National Engineering Laboratory for Animal Breeding, MOA Key Laboratory of Animal Genetics and Breeding, Department of Animal Genetics and Breeding, China Agricultural University, Beijing, China; 2 The State Key Laboratory of Animal Nutrition, Institute of Animal Sciences, Chinese Academy of Agricultural Sciences, Beijing, China; 3 Beijing General Station of Animal Husbandry, Beijing, China

## Abstract

The feed conversion ratio (FCR) is an essential economic trait for pig production, and is directly related to feed efficiency. Studies identifying the differential expression of functional genes involved in biological and molecular mechanisms in the intestine in relation to growth performance are rare. In this study, RNA-Seq was used to identify transcriptomes in caecal and colonic mucosal tissues in order to determine the differential expression of genes from two full-sibling pairs and two half-sibling pairs of Landrace finishing pigs with opposing FCR phenotypes. In total, 138 (comparison of high and low FCR in caecal mucosa), 64 (comparison of high and low FCR in colonic mucosa), and 165 (contrast between the caecal and colonic mucosa) differentially expressed genes were identified. Some of these genes were functionally related to energy and lipid metabolism, particularly short chain fatty acids metabolism, as well as gastrointestinal peristalsis and ion transport. Functional annotation were performed to identify differentially expressed genes, such as GUCA2A, GUCA2B, HSP70.2, NOS2, PCK1, SLCs, and CYPs, which may positively influence feed efficiency in Landrace pigs. These differentially expressed genes need to be further tested for candidate genes that are related to feed efficiency.

## Introduction

The performance of livestock animals is influenced by genetics and the environment, including diet and rearing conditions^1^. Feed accounts for more than 60% of the costs of pig production. Therefore, improving feed efficiency (FE) is an important method for reducing costs in the pig industry. FE can be measured using the feed conversion ratio (FCR). The FCR is the feed intake divided by the weight gained during a specified period. Thus, an animal with a high FCR value is less efficient at converting feed into body mass than one with a low FCR. Previous studies have indicated that the heritability values for FCR range from 0.13 to 0.31^2,3^.

Gene expression profiling in the liver and adipose tissue in pigs has revealed that the biological processes of lipid metabolism, mitochondrial activity, and glucose synthesis are associated with FE^4^. In cattle, differentially expressed genes (DEGs) between high and low residual feed intake (RFI) groups are related to cell growth and differentiation, lipid metabolism, and carbohydrate metabolism^5^.

Genetic lines of chickens with different feed efficiency have also been associated with changes in gut physiology and gut microbial composition^6^. There are a variety of ways through which the microflora can have a negative impact on the host, including the use of excessive amounts of energy, and the diversion of energy towards the immune system by inducing inflammatory responses^7^. Some nutrients, such as resistant starch (RS), cannot be digested completely and need to be fermented by caecal and colonic microbes to produce short-chain fatty acids (SCFAs)^8,9^.

In this study, we report, for the first time, a complete dataset detailing the transcriptome of caecal and colonic mucosal tissues identified from female near-market weight Landrace pigs with high or low FCR using RNA-Seq. We identified genes that were significantly differentially expressed between locations or groups to investigate whether they are associated with growth traits and/or microbial digestion. The putative candidate genes identified could lead to an improved understanding of digestion in the large intestine while providing new insights into growth traits.

## Results

### Overview of RNA-Seq data and differentially expressed genes analyzed

cDNA libraries were constructed for each RNA-Seq sample (n = 16), sequencing was conducted on an Illumina Hiseq. 4000 platform, and 150-bp paired-end reads were generated. As shown in Table 1, RNA-Seq analysis yielded between 48.72 and 77.40 million total clean reads per sample, and the percentage of mapped reads ranged from 69.24 to 73.02%. The percentages of mapped reads were similar between samples, and more than half were located within an exon, with percentages ranging from 58.72 to 69.85%. Other reads mapped within the untranslated region, introns, and intergenic regions, and only the uniquely mapped reads were analysed.

**Table 1:** RNA sequencing of mRNA from the caecal and colonic mucosa of Landrace female finishing pigs with high and low FCR.

Group	Sample	Total reads (million)	Total mapped reads (%)	Unique matched reads (%)	Multi matched reads (%)	CDS exons (%)	5′UTR exons (%)	3′UTR exons (%)	Intron (%)	Intergenic (%)
Hce	H1caecum	53.58	72.70	68.45	4.26	64.57	1.45	12.28	8.04	13.66
	H2caecum	52.68	71.05	66.83	4.22	69.85	1.73	10.91	5.52	11.99
	H3caecum	51.18	71.55	67.36	4.19	64.88	1.44	11.93	8.04	13.71
	H4caecum	58.62	71.04	66.91	4.12	58.72	1.05	12.76	11.57	15.91
Lce	L1caecum	61.19	72.94	68.69	4.25	67.88	1.68	11.45	6.44	12.55
	L2caecum	48.72	73.00	68.68	4.32	66.26	1.59	12.00	6.67	13.48
	L3caecum	66.97	72.90	68.62	4.28	68.15	1.67	11.26	6.36	12.56
	L4caecum	58.57	70.56	66.37	4.20	65.84	1.54	11.12	7.98	13.51
Hco	H1colon	69.50	71.48	67.12	4.36	68.89	1.72	11.04	6.00	12.35
	H2colon	77.40	70.15	65.82	4.33	69.16	1.77	10.53	6.22	12.32
	H3colon	69.08	70.01	65.71	4.30	64.42	1.54	10.86	9.06	14.13
	H4colon	54.37	73.02	68.90	4.12	65.32	1.38	12.35	7.42	13.54
Lco	L1colon	64.75	69.24	64.83	4.41	68.20	2.00	9.26	7.71	12.83
	L2colon	60.30	72.25	67.90	4.35	64.69	1.51	11.38	8.25	14.17
	L3colon	69.99	69.84	65.49	4.34	68.86	1.84	9.51	7.22	12.56
	L4colon	63.21	72.54	68.28	4.28	66.03	1.61	11.80	7.22	13.35

Uses *Sus scrofa* 10.2 as the reference genome annotation to classify the mapping tags to the different regions. Hce, caecal samples of HFCR group; Lce, caecal samples of LFCR group. Hco, colonic samples of HFCR group; Lco, colonic samples of LFCR group.

**Figure 1:**
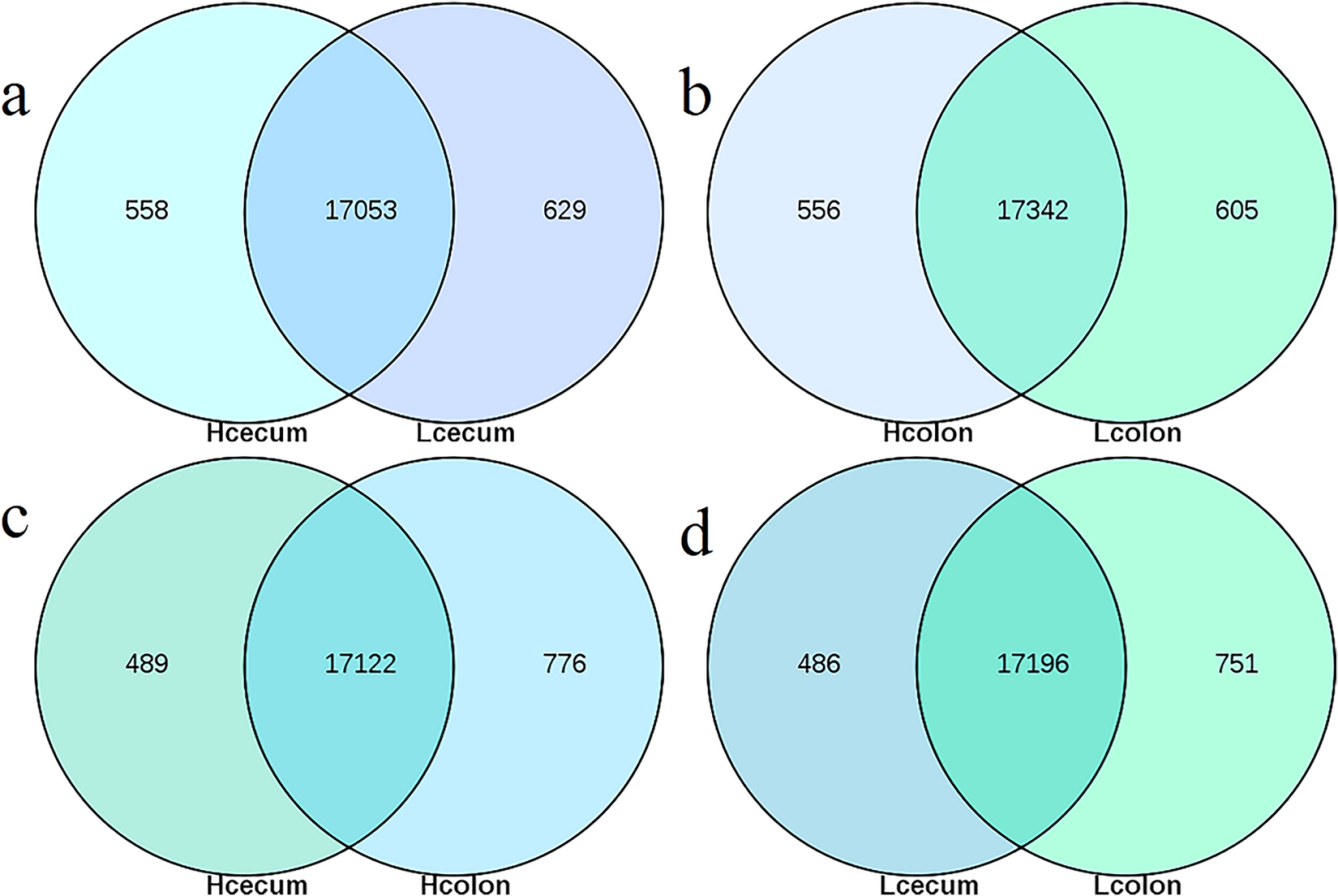
Venn diagrams representing gene expression in four groups of pigs. Levels of gene expression in the caecal and colonic mucosa of pigs with high or low FCR: Hcaecum and Lcaecum (**a**), Hcolon and Lcolon (**b**), Hcaecum and Hcolon (**c**), and Lcaecum and Lcolon (**d**) are shown. The total number of expressed genes in each group is shown. The number of common genes is shown in the overlapping segments.

Expression levels of all genes were calculated using the RPKM method and Tophat 2 software, and the results of all groups are presented in Supplementary Table S2. The total number of expressed genes in the caecum and colonic mucosa were similar between groups (17,611–17,947) in four cDNA libraries. Most genes were expressed in both groups, and there were more than 17,000 common genes in all four comparisons between the caecal and colonic mucosa in the high and low FCR pigs (Fig. 1).

**Figure 2:**
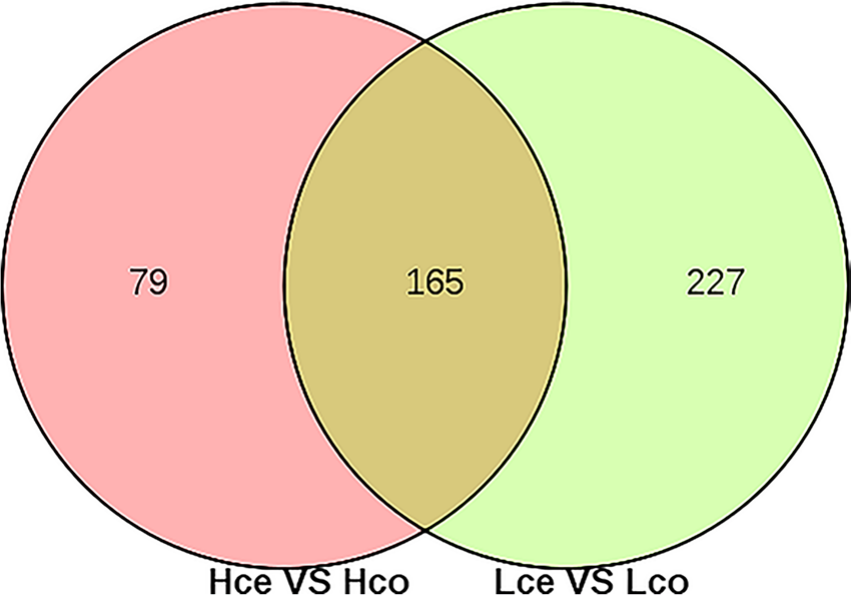
Venn diagrams showing DEGs between the caecal and colonic mucosa in the high and low FCR groups.

Differentially expressed genes (DEGs) were identified in each comparison, with criteria of a ≥2-fold difference and a q-value of less than 0.05 (|log2FC| ≥ 1, q < 0.05). The up- and down-regulated DEGs are listed for each comparison, and the details of all DEGs in the comparisons are shown in Supplementary Tables S3 and S4. In total, 138 genes were different in group caecal mucosa of high FCR compared with caecal mucosa of low FCR (Hce vs Lce), 64 genes were different in group colonic mucosa of high FCR compared with colonic mucosa of low FCR (Hco vs Lco), 244 genes were different in group caecal mucosa of high FCR compared with colonic mucosa of high FCR (Hce vs Hco), and 392 genes were different in group caecal mucosa of low FCR compared with colonic mucosa of low FCR (Lce vs Lco). Subsequently, 165 genes from the intersection of Hce vs Hco and Lce vs Lco were considered as the differentially expressed genes between caecal and colonic mucosal tissues (Fig. 2). Tables 2 and 3 list 40 and 13 annotated differentially expressed genes in caecal and colonic mucosa respectively, with either higher or lower expression in high FCR compared to low FCR pigs.

**Table 2:** List of 40 differentially expressed genes (DEGs) between caecal mucosa with high and low FCR.

Gene	Locus	FPKM_Hce	FPKM_Lce	Log2(Fold change)	q_value	Gene description
GUCA2A	6:156476367-156478316	1822.97	641.96	−1.51	6.16E-03	Guanylin
HSP70.2	GL896522.1:2-1799	1450.72	417.43	−1.80	3.40E-03	*Sus scrofa* heat shock protein 70.2
ISG12(A)	7:122215477-122220545	1395.64	417.83	−1.74	3.40E-03	interferon, alpha-inducible protein 27
UBD	7:25330260-25332807	1242.79	287.87	−2.11	3.40E-03	ubiquitin D
IGLV-11	14:52228869-52229344	1169.88	534.43	−1.13	1.52E-02	immunoglobulin lambda variable 11
S100G	X:13996225-14092759	1043.02	312.73	−1.74	6.16E-03	S100 calcium binding protein G
ASS1	1:304454128-304508998	877.10	234.95	−1.90	3.40E-03	argininosuccinate synthase 1
IGLV-2	14:52404074-52404828	564.54	191.16	−1.56	3.40E-03	immunoglobulin lambda variable 2
MS4A12	2:10736890-10753331	530.87	254.17	−1.06	1.90E-02	membrane spanning 4-domains A12
CLDN4	3:10776891-10780656	236.74	101.29	−1.22	3.40E-03	claudin 4
HBB	9:5632714-5634344	235.47	93.61	−1.33	3.40E-03	hemoglobin, beta
GZMB	7:79831196-79834605	204.96	97.37	−1.07	1.52E-02	granzyme B
UBE2L6	2:12987516-13002993	177.71	83.94	−1.08	3.40E-03	ubiquitin conjugating enzyme E2L 6
ISG15	GL895967.2:58448-59139	139.46	29.59	−2.24	3.40E-03	ISG15 ubiquitin-like modifier
GZMA	16:36385657-36393941	117.24	48.25	−1.28	3.40E-03	granzyme A
GNLY	3:61952961-61957608	114.36	52.31	−1.13	3.40E-03	antimicrobial peptide NK-lysin precursor
GBP2	4:139612346-139633862	112.97	44.49	−1.34	3.40E-03	guanylate binding protein 2, interferon-inducible
NOS2	12:46022453-46053026	103.76	35.61	−1.54	3.40E-03	nitric oxide synthase 2
SLC30A10	10:11615849-11627111	85.04	36.22	−1.23	3.40E-03	solute carrier family 30 member 10
ENTPD8	1:314009380-314012067	78.46	39.17	−1.00	3.40E-03	ectonucleoside triphosphate diphosphohydrolase 8
PLEKHG6	5:66793344-66811338	66.27	28.88	−1.20	6.16E-03	pleckstrin homology and RhoGEF domain containing G6
ANPEP	7:60240144-60262914	64.18	22.43	−1.52	1.10E-02	alanyl aminopeptidase, membrane
SLA-7	7:27613956-27617558	63.35	27.62	−1.20	3.40E-03	MHC class I antigen 7
KIFC2	4:415602-422897	58.43	24.88	−1.23	3.40E-03	kinesin family member C2
OAS1	14:41230164-41288614	58.41	25.10	−1.22	4.19E-02	2′-5′-oligoadenylate synthetase 1
7SK	7:134400748-134401079	54.67	22.78	−1.26	2.47E-02	7SK RNA
IGSF23	6:47050121-47057994	51.73	15.06	−1.78	3.40E-03	immunoglobulin superfamily member 23
PARP15	13:147507154-147531686	45.23	6.90	−2.71	3.40E-03	poly(ADP-ribose) polymerase family member 15
C4BPA	9:74195615-74207951	39.17	17.67	−1.15	3.74E-02	complement component 4 binding protein alpha
SLC14A1	1:105418611-105444533	37.42	8.04	−2.22	3.40E-03	solute carrier family 14 member 1
OASL	14:43414433-43431814	30.69	7.16	−2.10	3.40E-03	2′-5′-oligoadenylate synthetase-like
FRMD1	1:2657896-2672308	11.75	3.31	−1.83	3.40E-03	FERM domain containing 1
IGKV-3	3:59934903-59935620	110.74	732.09	2.72	3.40E-03	immunoglobulin kappa variable 3
IGLV-4	14:52392751-52393255	68.83	552.68	3.01	3.40E-03	immunoglobulin lambda variable 4
CH242-307A4.1	3:67957189-67973255	48.89	337.90	2.79	3.40E-03	lithostathine-like precursor
IGLV-5	14:52384767-52385271	27.55	171.00	2.63	3.40E-03	immunoglobulin lambda variable 5
PIGR	9:73889671-73899096	71.17	150.36	1.08	3.40E-03	polymeric immunoglobulin receptor precursor
REG3G	3:68078690-68081405	5.92	129.41	4.45	3.40E-03	regenerating islet-derived 3 gamma
C4BPA	9:74110908-74154259	45.63	104.31	1.19	3.40E-03	complement component 4 binding protein alpha
CHAC1	1:145522852-145525603	2.49	64.03	4.68	3.40E-03	ChaC glutathione specific gamma-glutamylcyclotransferase 1

**Table 3:** List of 13 differentially expressed genes (DEGs) between colonic mucosa with high and low FCR.

gene	locus	FPKM_Hco	FPKM_Lco	log2(fold_change)	q_value	Gene description
TRPV6	18:7636964-7652271	14.71	1.73	−3.09	3.40E-03	transient receptor potential cation channel subfamily V member 6
SPINK1	2:155635124-155640264	32976.60	10332.90	−1.67	3.40E-03	serine peptidase inhibitor, Kazal type 1
IGLV-2	14:52404074-52404828	303.47	112.61	−1.43	3.40E-03	immunoglobulin lambda variable 2
TMEM120A	GL894019.2:55427-61934	42.33	16.05	−1.40	3.40E-03	transmembrane protein 120A
CYP2B6	6:44929853-44978800	56.83	22.22	−1.35	1.71E-02	cytochrome P450 family 2 subfamily B member 6
FRMD1	1:2657896-2672308	33.53	14.14	−1.25	3.44E-02	FERM domain containing 1
CD209	2:71917404-71931695	24.01	55.44	1.21	3.40E-03	CD209 molecule
SLC2A13	5:73929816-74023533	39.91	105.25	1.40	3.40E-03	solute carrier family 2 member 13
CH242-307A4.1	3:67957189-67973255	3.18	9.41	1.56	1.32E-02	lithostathine-like precursor
IGLV-5	14:52384767-52385271	22.54	90.57	2.01	3.40E-03	immunoglobulin lambda variable 5
IGKV-3	3:59934903-59935620	39.11	169.95	2.12	6.16E-03	immunoglobulin kappa variable 3
IGLV-4	14:52392751-52393255	26.33	226.01	3.10	3.40E-03	immunoglobulin lambda variable 4
CHAC1	1:145522852-145525603	1.16	10.11	3.13	3.40E-03	ChaC glutathione specific gamma-glutamylcyclotransferase 1

### Functional annotation clustering of DEGs

Due to the limited annotation of the reference genome (*S. scrofa* 10.2), the DEGs were converted to human orthologs, and the online program Visualization and Integrated Discovery (DAVID) was used for gene ontology (GO) and Kyoto Encyclopaedia of Genes and Genomes (KEGG) pathway analysis. GO enrichment analyses contained ‘biological process’, ‘molecular function’, and ‘cell component’. Distinct functional separation between the DEGs and GO terms was observed through a graphical network drawn by ToppCluster software for each comparison. STRING was used to build protein-protein interactions nets of the DEGs encoding proteins.

**Figure 3:**
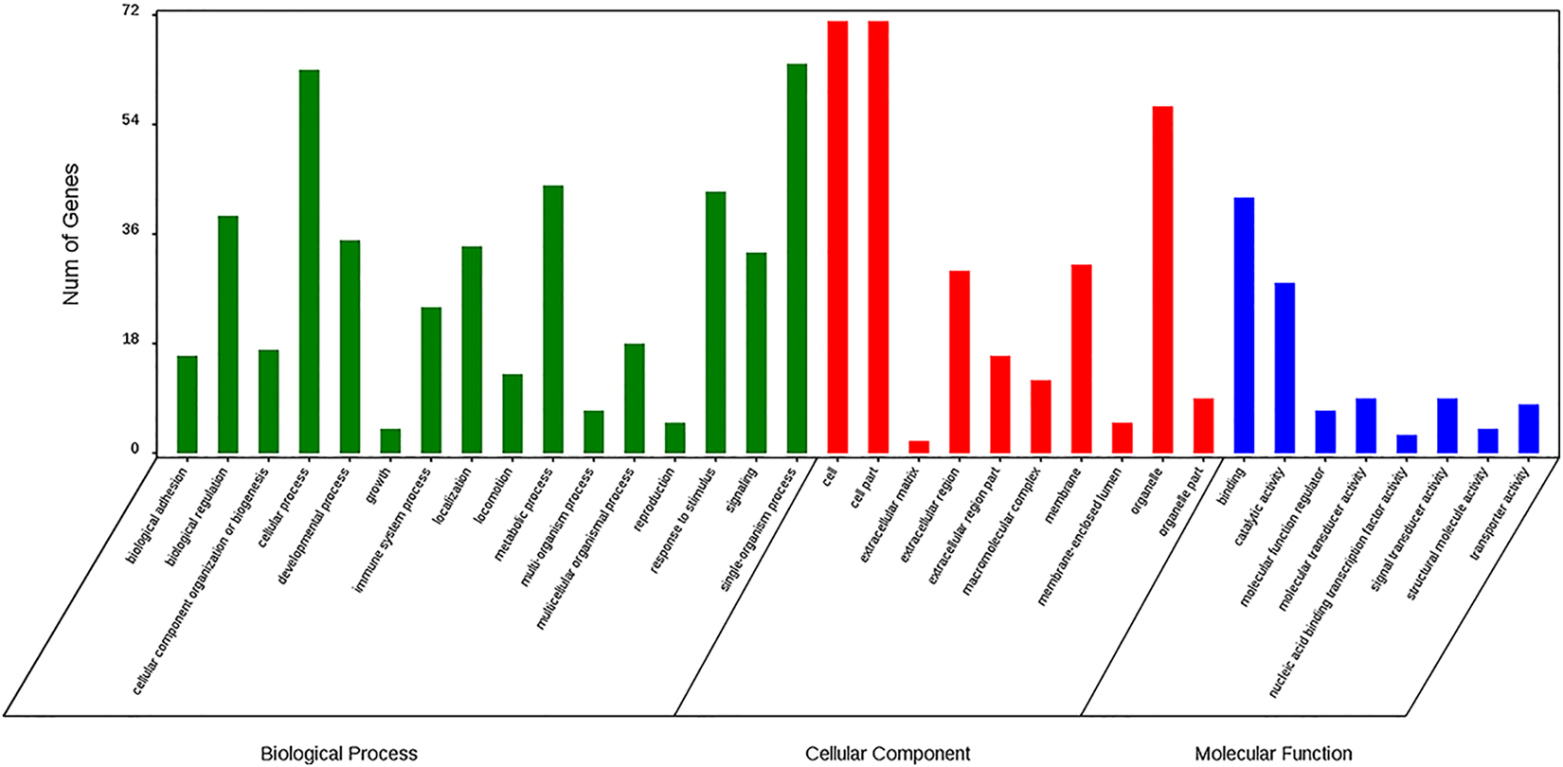
Enriched GO terms and the number of corresponding DEGs for each term in the Hce vs Lce group.

For GO enrichment and KEGG pathway analysis of DEGs between caecal mucosa in the high and low FCR groups, multiple significant pathways and GO terms are shown in Supplementary Table S5. The DEGs were primarily enriched in categories related to cellular processes, single-organism processes, biological regulations, metabolic processes, responses to stimuli, cells, cell parts, organelles, binding, and catalytic activity (Fig. 3). Furthermore, KEGG pathway analysis identified significant pathways that were mostly related to immunity and disease. Fatty acid metabolism was also mapped, but did not reach significance (*P* = 0.078).

**Figure 4:**
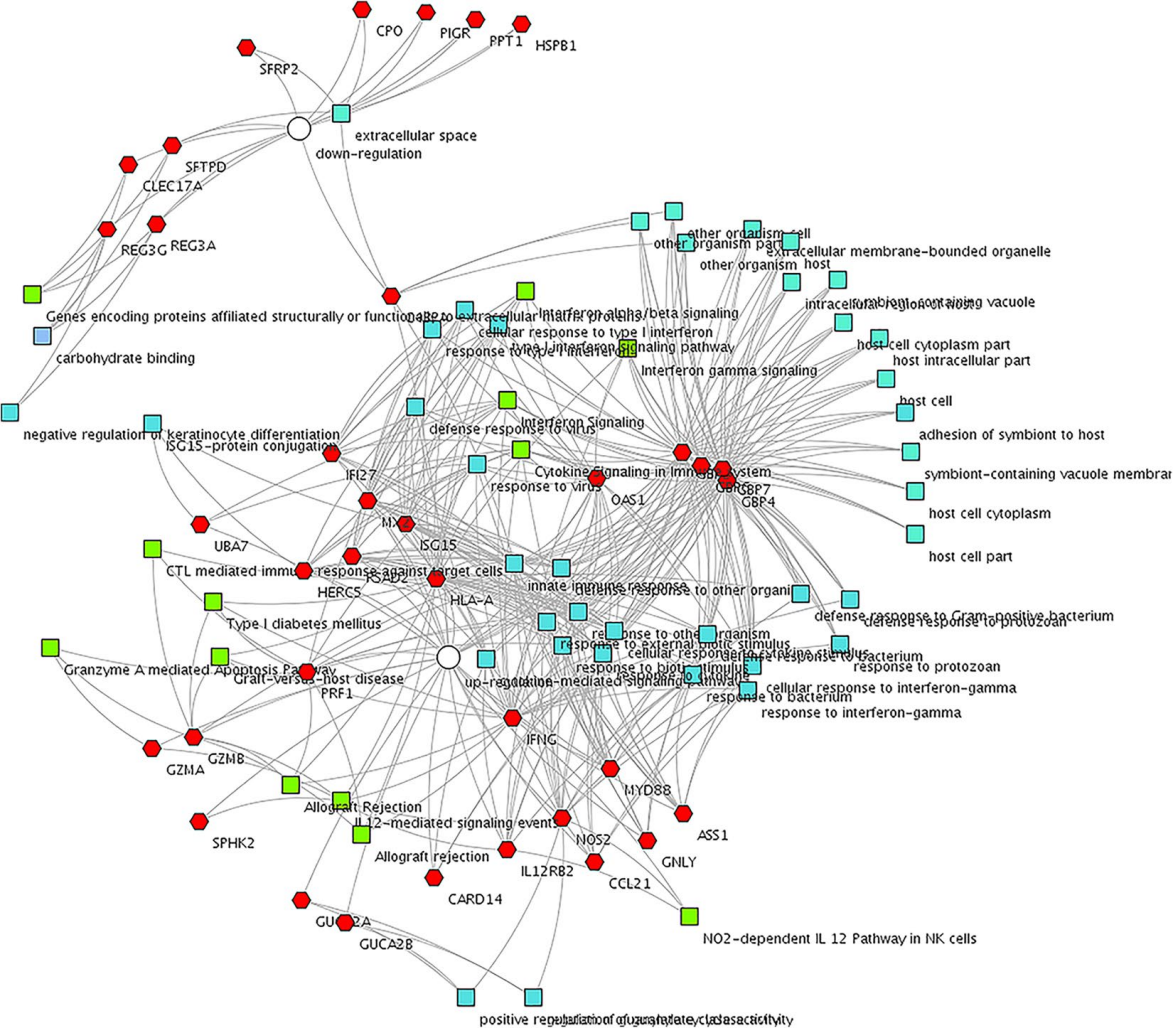
Relationship between DEGs, pathways, and GO terms of Hce vs Lce. Functional association analysis performed by ToppCluster based on pathway networks showing enriched terms from Gene Ontology and pathways. The top part of the figure depicts significant enrichments for up-regulated DEGs in Hce compared with Lce; the lower part depicts significant enrichments for down-regulated DEGs in Hce compared with Lce. Red hexagon: DEG; green square: pathways; blue square: biological processes; grey square: molecular functions.

The functional network for DEGs was generated by ToppCluster as shown in Fig. 4. Most terms and pathways for DEGs were in categories associated with immunity and disease, which is consistent with previous results obtained through DAVID. The down-regulated genes encoded proteins associated with structure or function. The up-regulated genes were mostly related to immunity and disease. We identified a gene, complement component 4 binding protein alpha (C4BPA), which was found in groups of both up-regulated and down-regulated genes. This was because there were two transcripts belonging to the C4BPA gene, with one classified as being up-regulated and another classified as being down-regulated, when comparing the Hce group to the Lce group. C4BPA has been demonstrated to be involved in the pathway of complement and coagulation cascades, while the C4BP (C4b-binding protein) is known as a soluble complement inhibitor in the classical pathway of complement systems, and is important for body homeostasis^10^.

**Figure 5:**
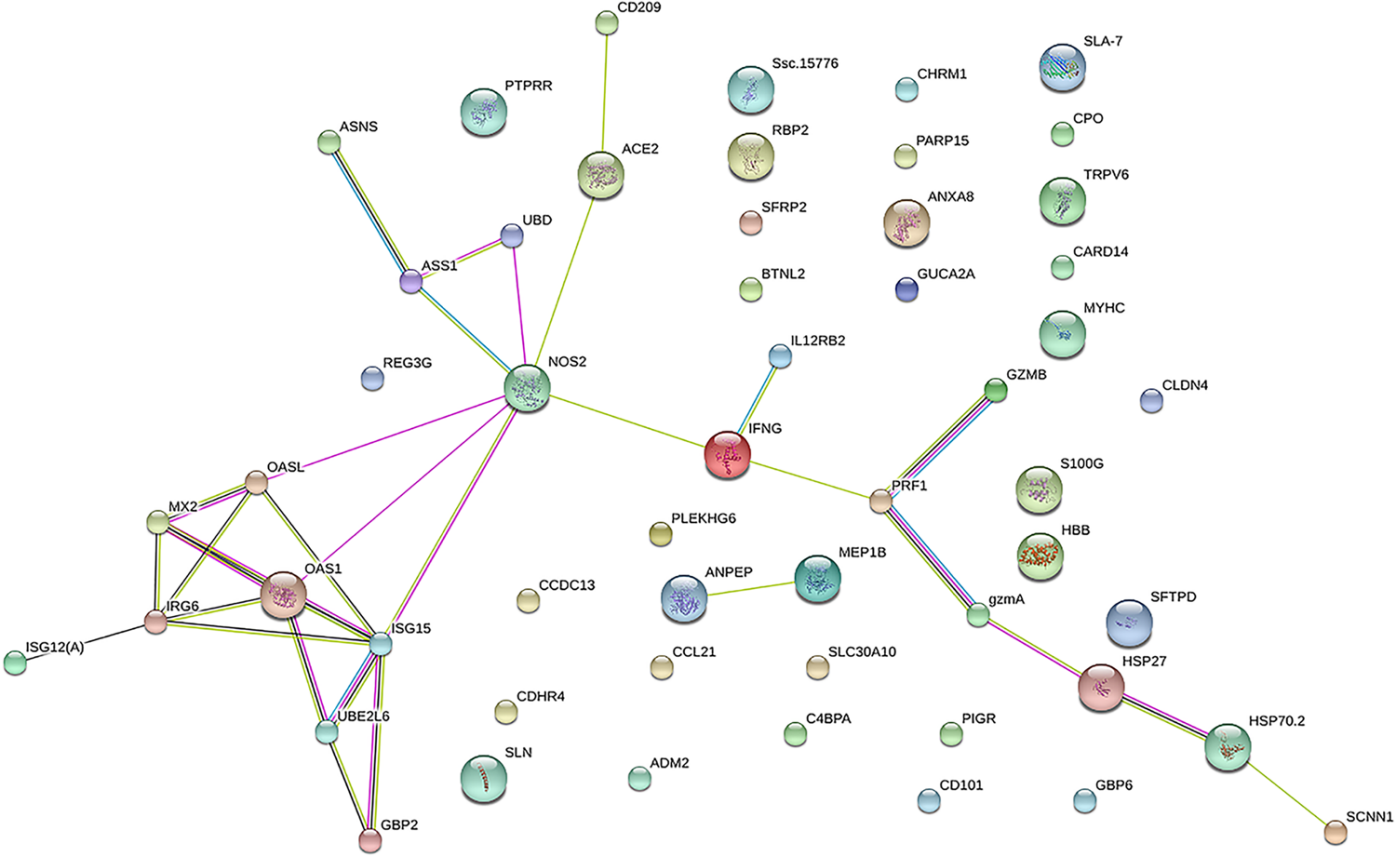
STRING analysis shows that DEGs are involved in known and predicted protein-protein interactions. STRING is used to analyse DEGs in the mucosa of Hce and Lce. The network nodes stand for those genes shown in Tables S3 and S4. Different coloured lines represent seven types of evidence used to predict associations. Red line: fusion evidence; green line: neighbourhood evidence; blue line: co-occurrence evidence; purple line: experimental evidence; yellow line: text mining evidence; light blue line: database evidence and black line: co-expression evidence.

A protein interaction network analysis was generated from the DEGs by STRING (Fig. 5), for Hce compared with Lce. The nitric oxide synthase 2 (NOS2) gene participated in multiple interactions in this network of the comparison. This gene encodes a nitric oxide synthase that is expressed in the liver, and its expression is induced by a combination of lipopolysaccharide and certain cytokines. The genes in this network were related to some signalling pathways, such as cytokine, interferon, interferon gamma, and citrulline and aspartate metabolism^11^.

DEGs in the colonic mucosa of the high and low FCR groups were functionally annotated, and were found to be related to cellular processes, single-organism processes, metabolic processes, cells, cell parts, organelles, binding, and catalytic activity (Fig. S2). Multiple significant GO terms are shown in Supplementary Table S6. Certain terms were significant and were related to metabolism (*P* < 0.05), such as cell recognition, ion transport (a biological process), beta-galactosidase activity, calcium channel activity, and carbohydrate binding (molecular function). Alpha-linolenic acid metabolism was mapped in the KEGG pathway, but did not reach significance (*P* = 0.097).

We found differences the common DEGs present in the caecal mucosa to those in the colonic mucosa in both high and low FCR groups. Subsequently, GO terms and KEGG pathways of DEGs were determined between the caecal and colonic mucosa. The primary GO terms clustered were cellular process, single-organism process, and metabolic process in ‘biological process’; cells, cell parts, and organelles in ‘cellular component’; and binding and catalytic activity in ‘molecular function’ (Fig. S3). Pathway analysis identified significant pathways that were mostly related to nutrient metabolism, and the PPAR signalling pathway was also mapped, reaching significance (*P* < 0.05) (Table S7).

Results for the DEG functional network are presented in Fig. S4, and pathways involving up-regulated DEGs were found to be associated with ascorbate and aldarate metabolism. The down-regulated DEGs were related to trans-membrane transport and drug metabolism. A protein interaction network of DEGs was also generated, and a few protein-coding genes were found to interact with others (Fig. S5). We found that forkhead box A2 (FOXA2) was associated with three other genes. This gene encodes a member of the forkhead class of DNA-binding proteins. These hepatocyte nuclear factors are transcriptional activators of liver-specific genes, such as albumin and transthyretin, and they also interact with chromatin. FOXA2 plays a pivotal role in regulating intestinal epithelial cell function^12^.

### DEGs compared with the QTL database

Using the Pig Quantitative Trait Locus (QTL) Database (Pig QTLdb), production and production-associated QTLs with a confidence interval of less than 5 Mb were selected^13^. Four DEGs in Hco vs Lco, 15 DEGs in Hce vs Lce, and eight DEGs in ce vs co were available (Table S8). The DEGs found in each comparison represented additional candidate genes affecting pig production.

## Discussion

In this study, systematic transcriptome profiling of caecal and colonic mucosal tissues from two full-sib pairs and two half-sib pairs of pigs with opposing FCRs was performed using high-throughput RNA-Seq technology. This could reduce false-positive results caused by genetic background noise and the number of replicates^13,14^.

The phenotypic trait, feed efficiency (FE), is an important economic trait for meat production in the pig industry. Thus, FE should be improved to reduce the costs of pig production. To date, FE can be measured by two highly correlated indicators, i.e., FCR and RFI, and a low FCR/RFI level signifies improvement in FE^15,16^. It is important to investigate the mechanisms of FE for pig breeding. Studies have suggested that energy metabolism in the liver and muscle tissues is essential for the regulation of FE of pigs^3,17,18^. Additionally, FE has also been related to the intestinal innate immune response and enhanced antimicrobial enzyme activity, and lower serum IL-8, myeloperoxidase and endotoxin levels have been found to be associated with high FE^19,20^.

Considerable colonisation of the intestine by microorganisms, especially in the large intestine, provides a homeostatic environment that affects the function of the gastrointestinal tract, disease resistance, health status, and animal performance^9,21^. The short-chain fatty acids (SCFA) produced in the large intestine by microbial fermentation of dietary fibre are rapidly absorbed from the large intestinal mucosa to provide the host with energy for basal metabolism^22^. Additionally, the caecal mucosa plays an important role in urea recycling in the gut of monogastric animals^23^.

In the study of caecal and colonic microbiota using the same animals^24^, Firmicutes and Bacteroidetes were the most abundant phyla in caecal and colonic microbiota of pigs in both groups, consistent with other studies^25,26^, and the dominant genera were *Prevotella* and *Bacteroides*. By comparing the high FCR and low groups in caecal and colonic microbiota respectively, the differentially predicted genes were most enriched in carbohydrate metabolism, followed by amino acid metabolism and energy metabolism.

*Prevotella* sp. *CAG:604* were the most different species in both low groups of caecal and colonic microbiota. *Prevotella* sp. *CAG:604* contains some genes that encode proteins involved in nutrient and energy metabolism, such as BN731_01873, ychF, gpmI, queF, speA, and fmt. The presence of fructo-oligosaccharides and starch in the lower intestine may cause a higher abundance of *Prevotella* ^27^. There were species of *Lactobacillus* enriched in the high group of both caecal and colonic microbiota, with *Lactobacillus* spp. often considered as probiotics. Several species of *Lactobacillus* belong to lactic acid bacteria (LAB), which convert carbohydrates to lactic acid in homofermentation or heterofermentation, or to acetic acid in heterofermentation^28^. The microorganisms actively participate in the process of material digestion in the lower intestine, and these nutrients are absorbed through the intestinal mucosa into the circulatory system. The genes expressed in the mucosa mainly maintain the healthy homeostasis of the intestinal environment, and some participate in the identification, catalysis and synthesis of substances.

We identified DEGs between pigs with different FCRs by profiling the caecal and colonic mucosa transcriptome, and identified nutrient metabolism pathways through GO and pathway analysis that were related to FE variation in pigs.

Previous studies of FE have identified many DEGs by RNA-Seq that are considered important for improving the growth of pigs^3,18^. In contrast to previous studies, the current study provides the first statistical analysis of growth traits to detect DEGs generated from intestinal mucosal tissue samples by RNA-Seq data. Based on transcriptome data, we compared the entire expression profile and analysed DEGs in caecal and colonic mucosal tissues in high and low FCR Landrace pigs. DEGs related to nutrient metabolism and growth, or to certain immune responses and diseases, may represent potential candidate genes that affect animal growth.

Although the top DEGs identified in the present study corresponded to immunity and disease, mucosal-associated bacterial communities help the host to displace pathogens, digest nutrients lost from the intestines, synthesise vitamins, and mature the immune system^29,30^. The mucosa is the first barrier involved in protecting the internal environment of the host. In addition, we identified several genes and pathways reported to be involved in important metabolic processes.

From the DEGs up-regulated in the caecal mucosa of the high FCR group compared with the low FCR groups, we identified several genes involved in growth traits (Table S3, Figs 3 and 4). Guanylate cyclase activator 2 A (GUCA2A) and guanylate cyclase activator 2B (GUCA2B) are related to ion and fluid homeostasis, irritable bowel syndrome (IBS), abdominal pain^31,32^, growth, and intestinal barrier integrity^33^, and were expressed in the gastrointestinal tract^34^. GUCA2A, GUCA2B, and GUCY2C were reported to bind to the GC-C signalling pathway via several steps, to sustain epithelial barrier integrity and affect IBD^35^. GUCA2B has advantages in the regulation of feeding, energy homeostasis, body mass, and metabolism^36,37^. In addition, GUCA2A binds to and increases the activity of guanylate cyclase, which is involved in glycogenolysis, and relaxation of smooth muscle. The gene GUCA2A also participates in biological processes of digestion by GO term annotation and GUCA2A and CUCA2B were involved in pathways of miscellaneous digestion events. As such, the higher expression of genes GUCA2A and CUCA2B could be beneficial for feed digestion and intestinal protection from external stimuli. Therefore, GUCA2A and GUCA2B might be good candidate genes for growth traits.

The results of other studies have suggested that the HSP70.2 (heat shock 70 kDa protein 1B) gene might be a candidate gene involved in obesity and type 2 diabetes^38,39^. As well as being involved in the immune system and cellular response, these genes are involved in ATP binding, ATPase activity and ATP metabolic processes, which provide the direct energy source for the body.

NOS2 is also considered to be a candidate gene, and controls gastrointestinal peristalsis^11^. By annotation of the GO term and pathway, NOS2 was found to be closely related to growth, as well as carboxylic acid binding, cAMP-dependent protein kinase regulator activity, cadherin binding in molecular function; responses to lipids, protein catabolic processes, nitrogen compound transport, responses to bacteria in biological processes; and pathways of arginine and proline metabolism. These activities are related to the metabolism of substances and indirectly affect the growth efficiency of animals.

Gene expression in caecal mucosa of high FCR group was compared with the low FCR group. Down-regulated genes were related to immunity and disease (Table 2), and up-regulated genes were connected with intestinal health and homeostasis. These genes (such as GUCA2A, HSP70.2, NOS2, ASS1, HBB, SLC30A10) can directly or indirectly affect nutrient absorption and utilization by animals, and therefore feed efficiency.

These different genes can be initially used as candidate genes to distinguish the difference in feed efficiency of pigs, although intensive research needs to be done in future to confirm this.

Within the up-regulated DEGs in the colonic mucosa of high FCR animals compared with that of the low FCR animals, the serine protease inhibitor Kazal type 1 referred to as pancreatic secretory trypsin inhibitor (SPINK1) that is known to inhibit trypsin and regulate homeostasis is secreted by the pancreatic acinar cells into the pancreatic juice^40^. This gene has been correlated with cancer growth and progression^41^.

Furthermore, high FPKM values were found in the caecal mucosa in both FCR groups. The cytochrome P450s 2B6 (CYP2B6) gene is involved in the synthesis of multiple drugs^42^, in the biotransformation of endogenous and exogenous compounds, and in the metabolism of many toxicants^43^.

There were not many different genes in the colonic mucosa of high FCR group compared with low group after we annotated these and removed genes of low expression. The down-regulated genes were also related to the immune system and disease, and interestingly, two genes IGLV-2 and FRMD1 were up-regulated, and five genes CH242-307A4.1, IGLV-5, IGKV-3, IGLV-4 and CHAC1 were down-regulated in both caecal and colonic locations.

Comparison of the caecal mucosa with the colonic mucosa in both the high and low FCR groups revealed differences between locations. Analysis of GO enrichment, KEGG pathway, protein-protein interactions, and QTL location (Tables S3, S8, Figs S3, S4), revealed that CYPs and solute carrier (SLC) gene families, and some metabolism-related genes, were up-regulated in the caecum compared with the colon. CYPs are of prime importance in the oxidation of unsaturated fatty acids and in catalysing metabolic reactions^44^. SLCs contain many glucose transporters, and are known as tumour suppressors^45^. SLCs directly facilitate the solute flux gradient, and indirectly power the solute electrochemical gradient^46^.

Phosphoenolpyruvate carboxykinase-1 (PCK1) was reported to participate in lipid metabolism^47^, is the rate-limiting enzyme in hepatic gluconeogenic pathways, and is closely related to obesity^48^. PCK1was also involved in pathways of glucose metabolism and pyruvate metabolism.

In summary, annotated DEGs in the caecal and colonic mucosal tissues of high and low FCR pigs were analysed and compared. The results revealed that the up-regulated DEGs related to loss of starch from the small intestine, some nucleotides, and drug metabolism. Up-regulated DEGs might also be directly or indirectly involved in FE regulation of pigs. Down-regulated DEGs were mostly related to immunity and disease in caecal mucosa or colonic mucosa tissues. GUCA2A, GUCA2B, HSP70.2, NOS2, PCK1, SLCs, and CYPs are possible candidate genes for FE in pigs. Overall, the current investigation utilizing RNA-Seq will add to the knowledge of gene expression in the pig intestine.

In conclusion, 138 (Hcaecum vs Lcaecum), 64 (Hcolon vs Lcolon), and 165 (caecum vs colon) differentially expressed genes were identified in the caecal and colonic mucosa of two landrace pig groups with diverging FCR. Some of these genes were functionally related to energy and lipid metabolism, in particular short chain fatty acids, as well as gastrointestinal peristalsis and biotransformation. GO enrichment, KEGG pathway, protein-protein interaction, and QTL location analysis were performed to identify DEGs, such as GUCA2A, GUCA2B, HSP70.2, NOS2, PCK1, SLCs, and CYPs, which may be potential candidate genes associated with growth traits in Landrace pigs. As only four pairs of pigs were used in this study, these results need be validated using a larger pig cohort in future. The findings of this study extend our understanding of the molecular mechanisms of feed efficiency that regulate growth trait in pigs.

## Methods

### Animals, phenotypes, and tissues

In this experiment, we used female Landrace pigs provided by Tianjin Ninghe primary pig breeding farm (Ninghe, China). Pigs (total 120) were housed in an environmentally controlled room (10 pigs in each pen), and given feed and water *ad libitum* throughout the experiment. Pedigree information was available for all pigs. Feed intake and body weight were recorded from 120 to 165 day old pigs using a Velos (Nedap co., LTD, Groenlo, the Netherlands) automated individual feeding system. The feed conversion ratio (FCR) was calculated for individuals for the 45 day trial period. The FCR values of all individuals were ordered, and high (20 pigs) and low (20 pigs) end individuals were tested using one way analysis of variance (ANOVA). The analysis found significant differences in FCR (see Supplementary Fig. 1), and two full-sib pairs and two half-sib pairs were selected in such a way that one sibling per pair had a low FCR and the other a high FCR (Table S1), in order to reduce the effects of genetic background as much as possible. Individuals with low feed efficiency and high FCR were classified as the low group, and those with high feed efficiency and low FCR were classified as the high group.

The selected pigs were euthanised in the morning on which they reached 166 days of age, and mucosal samples were scraped from the caecum and colon using glass microscope slides within 20 min of euthanisation. All samples were collected in sterile tubes and stored in liquid nitrogen until further analyses.

All experimental protocols were approved by the Animal Welfare Committee of China Agricultural University (permit number: DK996). Pigs were slaughtered in accordance with the approved slaughtering guidelines (GB/T 17236-2008) from the Quality Supervision, Inspection, and Quarantine Committee of the People’s Republic of China^13^. All efforts were made to minimize animal suffering during the study. Intestinal mucosal tissue of the caecum and colon were isolated aseptically and frozen in liquid nitrogen immediately after slaughter until required for RNA isolation^30^.

### RNA isolation

Total RNA was extracted from the caecal and colonic mucosa using Trizol (Takara Biotechnology Co. Ltd., Beijing, China) and following the manufacturer’s instructions. RNA integrity was checked using 1% agarose gel electrophoresis and the RNA concentration was measured with an Agilent 2100 Bioanalyser (Agilent, Santa Clara, CA).

### Library preparation and RNA sequencing

A total amount of 1.5 μg RNA per sample was used as input material to prepare RNA samples. Sequencing libraries were generated using NEB Next^®^ Ultra^TM^ RNA Library Prep Kit for Illumina^®^ (NEB, USA) following the manufacturer’s instructions, and index codes were added to attribute sequences to each sample. Briefly, mRNA was purified from total RNA using poly-T oligo-attached magnetic beads. Samples were fragmented using divalent cations under elevated temperature in NEB Next First-Strand Synthesis Reaction Buffer (5X). First-strand cDNA was synthesised using random hexamer primers and M-MuLV Reverse Transcriptase (RNaseH-). Second-strand cDNA synthesis was subsequently performed using DNA Polymerase I and RNase H. Remaining overhangs were converted into blunt ends via exonuclease/polymerase activities. After adenylation of 3′ ends of DNA fragments, NEB Next Adaptors with hairpin loop structure were ligated to prepare for hybridization. To select cDNA fragments with the appropriate length, the library fragments were purified with the AMPure XP system (Beckman Coulter, Beverly, USA). Subsequently, 3 μL USER Enzyme (NEB, USA) was used with size-selected, adaptor-ligated cDNA at 37 °C for 15 min followed by 5 min at 95 °C before PCR. Following this, PCR was performed using Phusion High-Fidelity DNA polymerase, Universal PCR primers, and Index (X) Primers. Finally, products were purified (AMPure XP system) and library quality was assessed on the Agilent Bioanalyser 2100 system.

The libraries were sequenced on an Illumina HiSeq platform, and 150 bp paired-end reads were generated. Sequenced RNA-Seq data for the caecal and colonic mucosa of 16 female Landrace pigs were available from NCBI Sequences Read Archive, under accession numbers SRP065563 and SRP065827.

### Read mapping on the *Sus scrofa* reference genome

Before reads could be mapped on the reference genome, the raw data had to be quality controlled. Clean reads were obtained using the software FASTQC, which removed reads containing adapter sequences, reads with poly-N sequences (in which the percentage of unknown sequences “N” was greater than 10%), and low quality reads (threshold quality score < 20, the percentage of read bases whose error rate was less than 1%). The *S. scrofa* genome was downloaded from the Ensembl database (ftp://ftp.ensembl.org/pub/release-85/fasta/sus_scrofa/dna/) as the reference genome for assembly using Tophat v2.1.1 software^49^. The number of mapping reads of exons, introns, and intergenic positions was calculated using RSeQC (version 2.6.3) in the genome^50^.

### Differential gene expression analysis

Cufflinks (version 2.2.1) was used for transcriptome assembling, and the Cuffmerge script from Cufflinks was used to merge the transcript files into a single transcriptome annotation. Differential gene expression was then analysed by Cuffdiff^49^. The expression of each gene was represented by counting the number of fragments per kilobase exon per million fragments mapped (FPKM value). Finally, |log2 (fold change)| ≥ 1 and q ≤ 0.05 were set as the threshold for differentially expressed gene (DEG) selection^49^.

### DEGs functional annotation clustering

DEGs were converted to human homologous Ensembl Genes and then utilised for functional enrichment analysis^51^. GO and pathway enrichment analysis of DEGs was implemented using the DAVID Bioinformatics Resources v6.8 (http://david.abcc.ncifcrf.gov/)^52,53^. P-values < 0.05 were deemed to show significant enrichment by DEGs. Figures for DEG GO enrichment were drawn online using Omicshare tools (http://www.omicshare.com/forum/). The protein-protein interaction network was analysed using STRING (http://www.string-db.org/)^54^. ToppCluster was used to create the potential important network(s) of pathways and DEGs; a P-value cut-off of 0.05 was selected for ToppCluster analysis^55^.

### DEGs comparison with the animal QTL database

QTL mapping of DEGs was performed by comparative analysis of DEGs and porcine QTL chromosome positions, which were collected from the animal QTL database (http://www.animalgenome.org/QTLdb). Furthermore, the DEGs mapped to metabolism QTLs were refined^56^.

## Electronic supplementary material

Dataset 1

Dataset 2

Dataset 3

Dataset 4

Dataset 5

Dataset 6

Supplementary Information
