# Supplementary material for: Differentially expressed genes in the caecal and colonic mucosa of Landrace finishing pigs with high and low food conversion ratios
Source: Sci Rep. 2017 Nov 2;7:14886. doi: 10.1038/s41598-017-14568-6 (PMC5668291; doi:10.1038/s41598-017-14568-6)
Supplement: Supplementary file 7 — Supplementary Information [file 41598_2017_14568_MOESM7_ESM.pdf]

Differentially expressed genes in the caecal and colonic mucosa of landrace finishing pigs with high and low food conversion ratio

Zhen Tan[1], Yuan Wang[1], Ting Yang[1], Kai Xing[1], Hong Ao[2], Shaokang Chen[3], Fengxia Zhang[1], Xitong Zhao[1], Jianfeng Liu[1], Chuduan Wang[1*]

[1]National Engineering Laboratory for Animal Breeding, MOA Key Laboratory of Animal Genetics and Breeding, Department of Animal Genetics and Breeding, China Agricultural University, Beijing, China

[2]The State Key Laboratory of Animal Nutrition, Institute of Animal Sciences, Chinese Academy of Agricultural Sciences, Beijing, China

[3]Beijing General Station of Animal Husbandry, Beijing, China

* Corresponding author; Email: cdwang@cau.edu.cn (CDW)

Supplementary Figure S1 Feed Conversion Ratio (FCR) calculated in high and low groups.

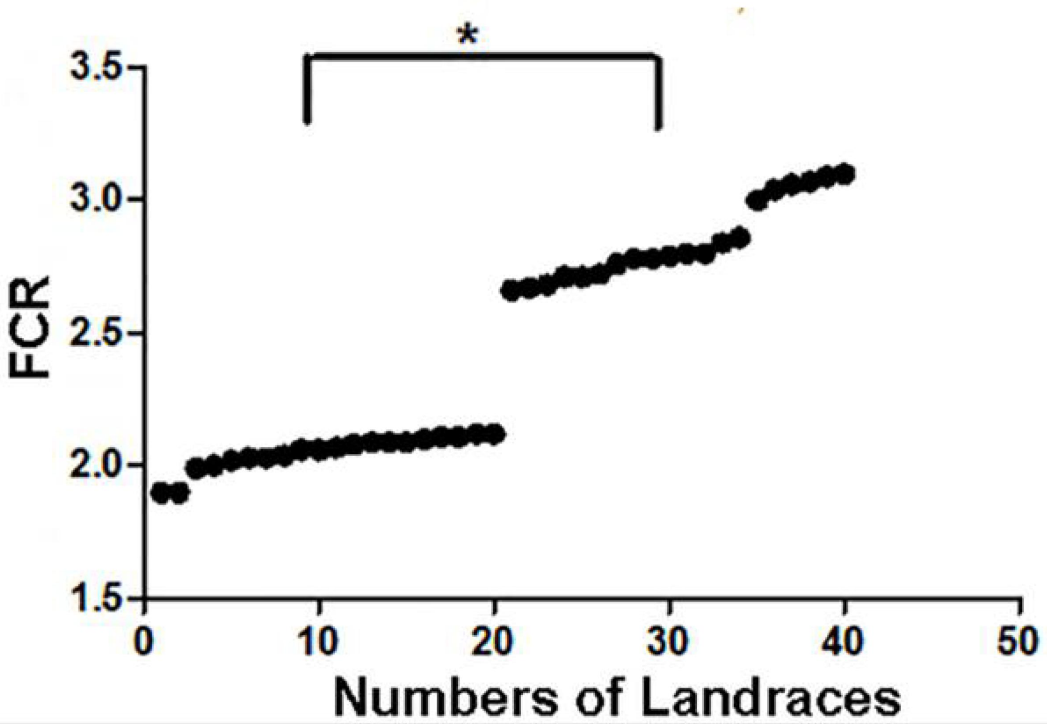

Supplementary Figure S2 Enriched GO terms and corresponding number of DEGs for each term in the high FCR group compared to low group of colon.

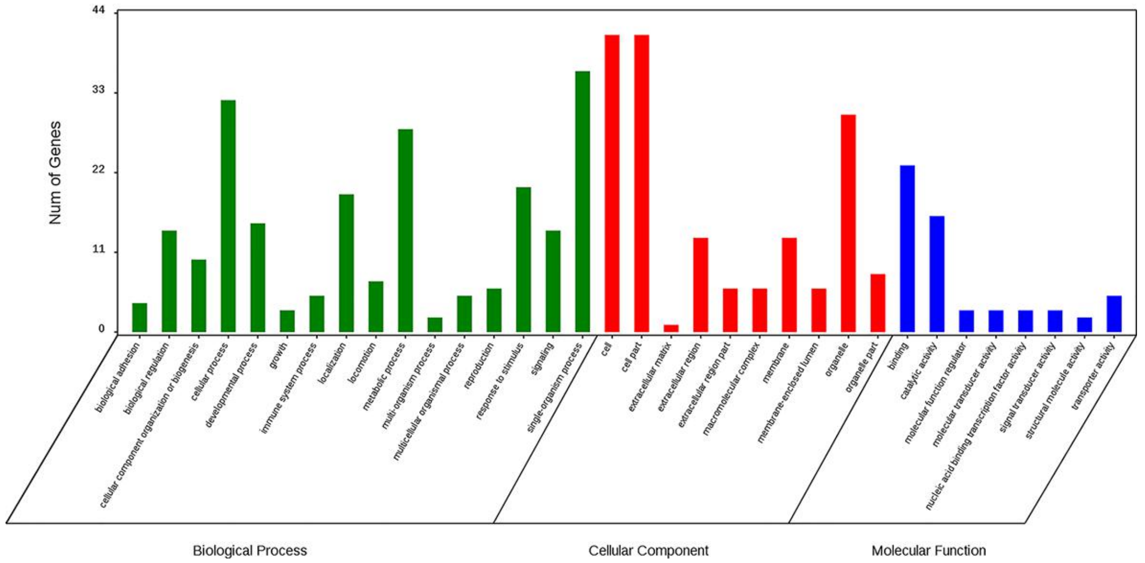

Supplementary Figure S3 Enriched GO terms and corresponding number of DEGs of each term for caecal mucosa group compared to colonic.

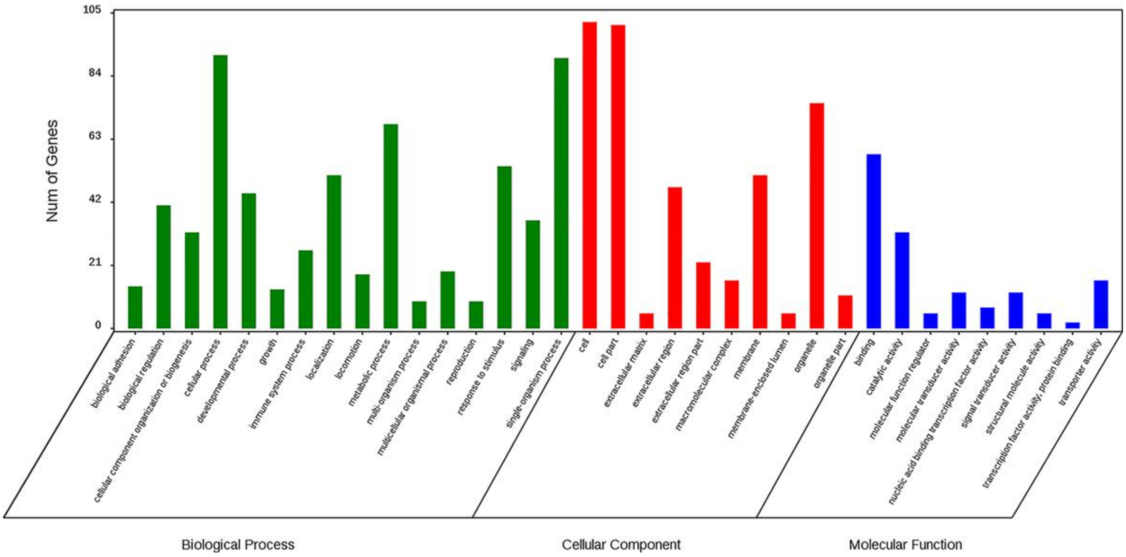

Supplementary Figure S4 Relationship between DEGs, pathways, and GO terms of caecal mucosa compared to colonic mucosa.

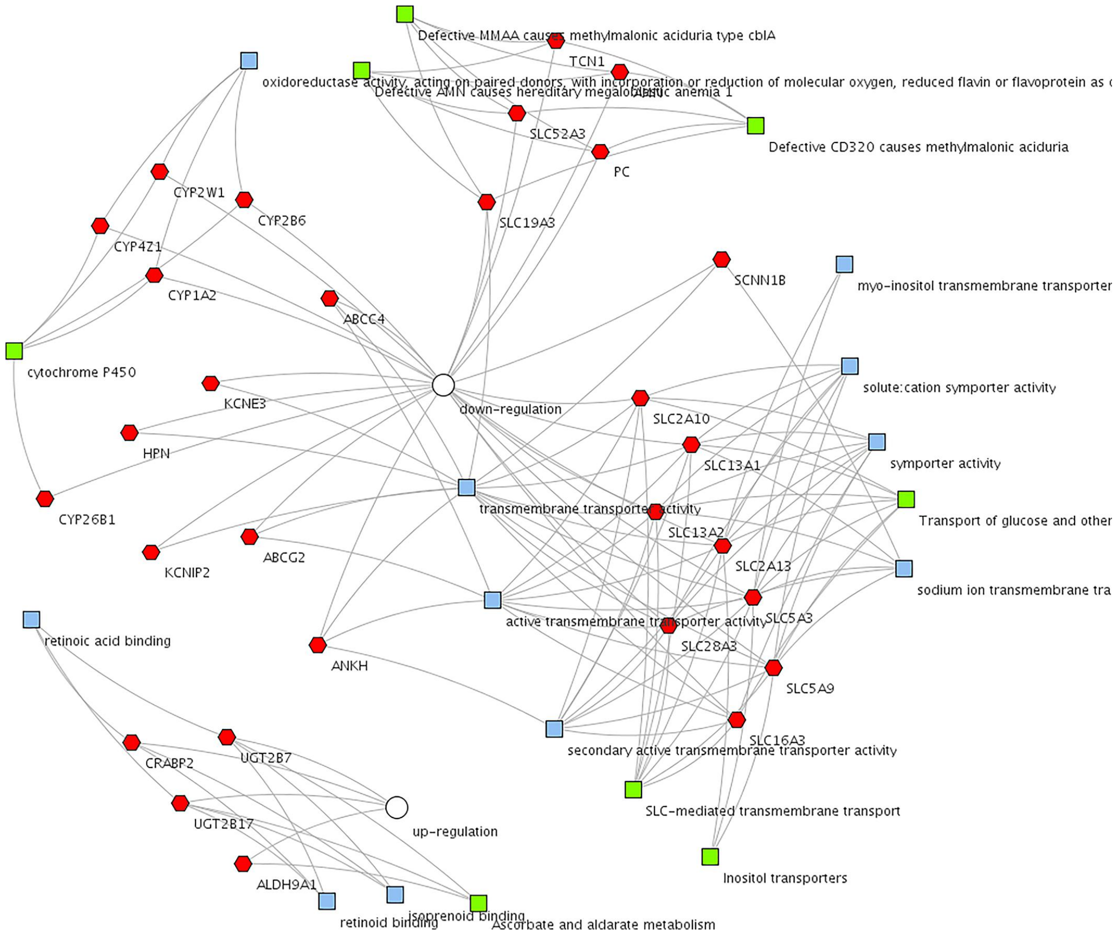

Supplementary Figure S5 STRING analysis showing that DEGs are involved in known and predicted protein-protein interactions in the caecal mucosa compared with the colonic mucosa.

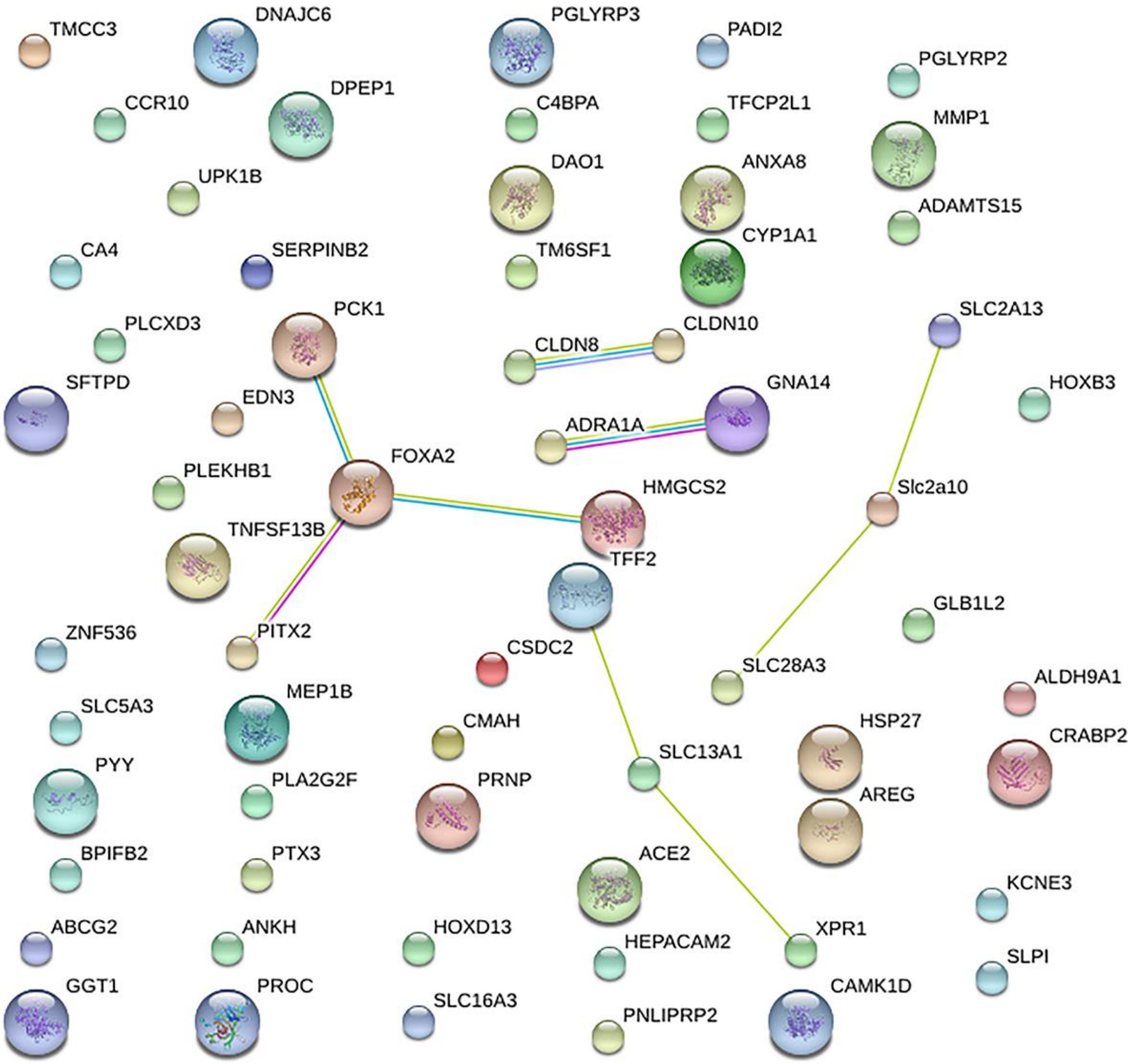

Supplementary Table S1 Individuals selected for this study.

ID	Status	Group	Sample	Initial BW (Kg)	Final BW (kg)	FCR
124110	Half-si	LFCR	L1	50.6	81.8	3.12
143014	b	HFCR	H1	49.4	92.5	2.02
144013	Half-si	LFCR	L2	50.1	86.2	2.8
143106	b	HFCR	H2	50.5	93.9	1.99
126614	Full-si	LFCR	L3	51.5	86.8	2.67
126606	b	HFCR	H3	50.2	92.5	2.03
130506	Full-si	LFCR	L4	49.7	84.3	2.79
130504	b	HFCR	H4	51.5	92	2.09

Supplementary Table S8.Common traits compared with the QTL database.

Comparison	Gene symbol	Chromosome	Gene Start (bp)	Gene End (bp)	Traits related to production_association OTLs
Hco vs Lco	CDO1	2	124815022	124828122	average daily gain;daily feed intake;feed intake;body weight
	CHRM1	2	8210976	8225289	average daily gain;body weight;bone mineral content;feed intake;
	GIF	2	11108689	11126311	feed intake;average daily gain;body weight
Hce vs Lce	SARDH	1	307202692	307268119	average daily gain;body weight
	PTPRR	5	37376180	37645956	ulna length
	IFNG	5	35374323	35379577	scapula length;body weight
	FAM46B	6	77873689	77881717	average daily gain
	TSPO2	7	41649695	41650796	average daily gain;days to 100 kg;body weight
	SLN	9	40309461	40314414	body weight
	MYH3	12	58110959	58132945	body weight
	KIFC2	4	415603	422897	average daily gain
	CHRM1	2	8210976	8225289	body weight
	CLDN4	3	10776892	10780656	body weight
	GBP6	4	139280329	139299776	feed conversion ratio
	GBP5	4	139413791	139425247	feed conversion ratio
	GBP2	4	139612347	139633862	feed conversion ratio
	MS4A12	2	10736891	10753331	body weight
	MYZAP	1	126414879	126509527	body weight
	CCDC13	13	28893092	28927988	residual feed intake
ce vs co	DNAJC6	6	135397188	135560153	daily feed intake
	EDN3	17	66846229	66869088	average daily gain;body weight
	GNA14	1	257106371	257310461	body weight
	PCK1	17	65094783	65100751	average daily gain;body weight
	PLEKHB1	9	8856572	8869589	daily feed intake
	SLC35F2	9	40366110	40418240	body weight
	TPPP3	6	25312514	25316150	body weight
	UPK1B	13	150294290	150320054	average daily gain

Hce, caecal samples of high FCR group; Lce, caecal samples of low FCR group. Hco, colonic samples of high FCR group; Lco, colonic samples of low FCR group. ce, caecal samples of all individuals, co, colonic samples of all individuals.
